# Self-Expanding Metallic Stents for Palliation of Esophageal Cancer: A Single Center Experience From Saudi Arabia

**DOI:** 10.7759/cureus.32096

**Published:** 2022-12-01

**Authors:** Adnan Alzanbagi, Laeeque A Qureshi, Ishtiaq Ahmed, Abdulaziz Tashkandi, Mohammed Khan, Ghaidaa A Alhazmi, Mohammed K Shariff

**Affiliations:** 1 Digestive and Liver Center, King Abdullah Medical City, Makkah, SAU; 2 Digestive and Liver Center (DLC) and Advanced Endoscopy Center, King Abdullah Medical City, Makkah, SAU; 3 Pharm. D, King Abdullah Medical City, Makkah, SAU

**Keywords:** cancer, gastroenterology, palliation, self-expanding metallic stent, ec- esophageal cancer

## Abstract

Background: Self-expanding metallic stents (SEMSs) are increasingly used as a non-surgical alternative for the palliation of advanced esophageal cancer (EC). However, there is a scarcity of real-life experience with the use of these stents exclusively in EC. The aim of this study is to evaluate the efficacy of SEMS in inoperable ECs in the western region of Saudi Arabia.

Methods: A retrospective review of SEMS placed in a tertiary referral hospital for histologically proven inoperable EC from 2016 to 2019. Demographics data, procedure success, complication, re-intervention, and mortality were analyzed.

Results: Forty-eight SEMS placed in 35 patients for palliation of dysphagia. The median age of patients was 68 years (range 31-95). 69% (24) patients have a lower third of EC and the rest have a middle third. SEMSs were placed successfully in all cases with symptomatic improvement. No major stent-related complication was seen. 28% (13) patients required re-intervention with additional SEMS placement, nine of which were for tissue in growth and four for distal migration. Median survival was 114 days (range 30-498). Most of the complications seen in fully covered SEMS compared to the partially covered 50% (8/16) vs 17% (5/30), respectively, p = 0.04. Chemo and/or radiotherapy were given to 51% (18) of the patients without any significant benefit on survival (p = 0.79) or re-intervention rate (p = 0.47) compared to those who did not.

Conclusion: SEMS is effective in palliating dysphagia in inoperable EC without major complications. Rates of tumors in growth and migration were comparable to other studies. SEMS provides long-term palliation.

## Introduction

Esophageal cancer (EC) is the sixth most common cause of cancer-related death globally with less than 10% surviving at five years [1,2]. Patients often present with incurable diseases and palliation of symptoms remains the main goal of therapy. Dysphagia is one of the most common symptoms requiring palliation to maintain nutrition. A number of palliative options like chemotherapy, radiotherapy, surgery, and thermal ablation are available to palliate dysphagia. Endoscopic placement of stents is now widely used to restore patency of the esophagus and improve dysphagia, as it is more efficacious with fewer complications compared to the above therapies [3].

Self-expanding metallic stents (SEMSs) are the most frequently used stents, as they require no prior dilatation and can be deployed using small-diameter delivery systems [4]. In more than 95% of cases, SEMS can be placed successfully with immediate improvement in dysphagia [5]. However, recurrent dysphagia due to tissue in- or overgrowth, food bolus, or migration is common and seen in more than 50% of patients requiring re-intervention [6]. Apart from dysphagia perforation, bleeding and fistulization are other recognized complications. Two types of SEMS are currently used, partially and fully covered. Partially covered has the advantage of reducing the risk of migration at the cost of in/overgrowth of tissue, whereas fully covered has the benefit of reduced in/overgrowth of tissue with higher migration risk [7]. Concomitant use of radiation with SEMS has raised concerns about increasing the rate of complications, however, the literature is conflicting [8].

Most of the data on the use of SEMS comes from studies that looked at all types of malignant esophageal strictures, including external compressions, fistulas, and tracheobronchial malignancy [9]. In addition, techniques used for SEMS insertion varied within the study. Techniques included inserting SEMS with and without prior dilatation and using fluoroscopy exclusively or a combination of endoscopy and fluoroscopy ADDIN EN.CITE.DATA [1-4,6-21]. Hence, the aim of this study is to retrospectively look at the outcome of SEMS use exclusively in primary EC, with the procedure performed using the standardized technique in a single tertiary referral center located in the Kingdom of Saudi Arabia, and characterize the demographics, complications, and survival.

This article was previously presented as a meeting abstract at the 2021 Clinical Gastroenterology Meeting on September 9, 2021.

## Materials and methods

Patients, design, and setting

The study was carried out in the department of Digestive and Liver Disease & Advance Endoscopy Center of King Abdullah Medical City (KAMC) located in Makkah Region, Saudi Arabia. KAMC is a tertiary referral center for the whole of the western region of KSA. All patients with inoperable ES who had SEMS inserted were identified from the hospital endoscopy database, starting from January 2010 till May 2015. The hospital patient records were retrospectively reviewed and information about age, gender, lesion locations, pathological types, type of therapy received (chemo/radiotherapy), stent length, post-procedural complications, improvement in dysphagia, and 30-day mortality were collected. The study was approved by the institutional review board of KAMC.

Definition

Technical success - placement of stent accurately across the EC as documented both fluoroscopically and endoscopically. Improvement in dysphagia - ability to swallow both liquid and solid food following stent placement. Bleeding - any gastrointestinal (GI) bleeding during stent insertion that required endoscopic intervention or GI bleeding reported as melena or hematemesis following stent insertion. Perforation - any evidence of a tear in the lining of the GI tract documented by either radiology or endoscopy. Migration - displacement of stent following appropriate stent insertion as documented by either radiology or endoscopy. Re-intervention - placement of a second stent. Mortality - whether a patient was alive or dead at 30 days following stent insertion.

Endoscopic stent insertion

**Figure 1:**
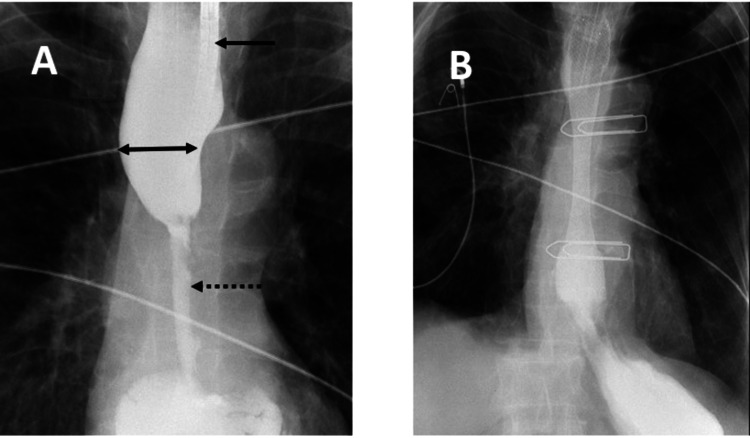
Fluoroscopy with gastrograffin at the time of upper endoscopy (A) There is a long segment distal esophageal luminal narrowing (dashed arrow) suggestive of esophageal cancer with proximal dilatation of the esophagus (double head arrow) with gastrograffin hold up and endoscope (solid arrow) in place. (B) Post stent insertion. Stent in situ with free flow of gastrograffin to stomach.

All the SEMSs inserted were either Evolution controlled release (Cook, Limerick, Ireland) or WallFlex (Boston Scientific, Galway, Ireland) esophageal stents which included either fully or partially covered depending on the choice of the endoscopists. All procedures were done under sedation and fluoroscopic guidance. The endoscope was introduced to the site of stricture and a guidewire was carefully maneuvered through the stricture. Location, a proximal, distal end, and the length of the stricture were noted using both endoscopic and fluoroscopic visualization (Figure 1A). With the guidewire in place, the endoscope was removed, and the SEMS deployed under fluoroscopic guidance. The endoscope was reintroduced to confirm the accurate placement of the proximal end of the SEMS, no attempt was made to pass the scope through the SEMS. The contrast was injected to delineate any evidence of perforation fluoroscopically (Figure 1B). No prior dilatation of the stricture was done.

Statistics

Numerical variables are expressed as median with range. Categorical variables as percentages. Complications between subgroups were compared using χ² and survival difference by using Mann-Whitney Rank Sum.

## Results

Forty-eight SEMS were placed in 35 patients, and all were placed for palliation of dysphagia. The median age of patients was 68 years (range 31-95) with 46% (16) males and the rest females. The EC was located in 69% (24) of cases in the lower third of the esophagus and the rest in the middle third. 77% (27) of these EC were adenocarcinomas and the remaining 23% (8) were squamous carcinomas. There were no stents inserted in the upper third of the esophagus. 89% (31) of these were inoperable due to advance (Stage T4 with or without metastasis) disease and 11% (4) were unfit for surgery. Of the 46 SEMS placed, 65% (30) were partially covered and the rest, 35% (16) were fully covered. 51% (18) of patients received chemo and/or radiotherapy (Table 1).

**Table 1:** Study patient’s characteristics

Characteristics	Number = 35 (percentage)
Age: median (range)	68 (31-95)
Gender: male: female	0.8:1
Oesophageal Cancer Location	
Upper	0 (0)
Middle	11 (31%)
Lower	24 (69%)
Oesophageal cancer type	
Adenocarcinoma	27 (77%)
Squamous carcinoma	8 (23%)
Received Chemo/Radiotherapy	
Yes	18 (51%)
No	17 (49%)

Technical success and complications

All the SEMS placed were technically successful leading to symptomatic improvement in dysphagia and all patients were able to take both liquid and solid food following the stent placement. Recurrent dysphagia was reported in 34% (12) of the patients post SEMS placement and required further intervention. One of these patients had re-intervention twice, hence a total of 13 SEMSs were reinserted in 12 patients. 20% (9) required re-interventions due to tissue in/overgrowth and 9% (4) for stent migration. There were no stent-related complications in the form of bleeding, perforation or death (Table 2).

**Table 2:** Complications of SEMS

Complications	Patients, n (%)
Perforation	0 (0%)
Bleeding	0 (0%)
Tumor in/over growth	9 (20%)
Stent migration	4 (9%)
Death	0 (0%)

**Figure 2:**
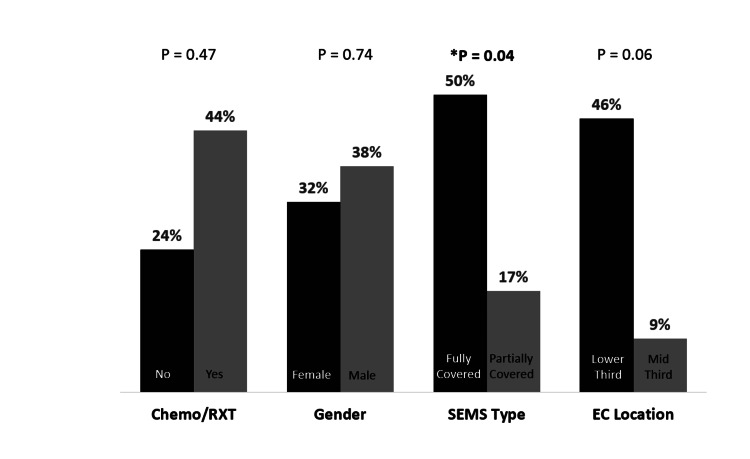
Reintervention rates depending on chemo-radiotherapy, gender, stent type and tumor location

Placement of a fully covered SEMS carried significantly higher rates of complications compared with partially covered SEMS (50% (8/16) vs 17% (5/30), respectively, p = 0.04). Depending on the location, EC of lower third of esophagus showed a trend toward more complications in comparison to middle third, this though did not reach the level of statistical significance (46% [11/24] vs 9% [1/11], p = 0.06). Having chemotherapy and/or radiotherapy nor the gender type were associated with excess complications (Figure 2).

Survival

**Figure 3:**
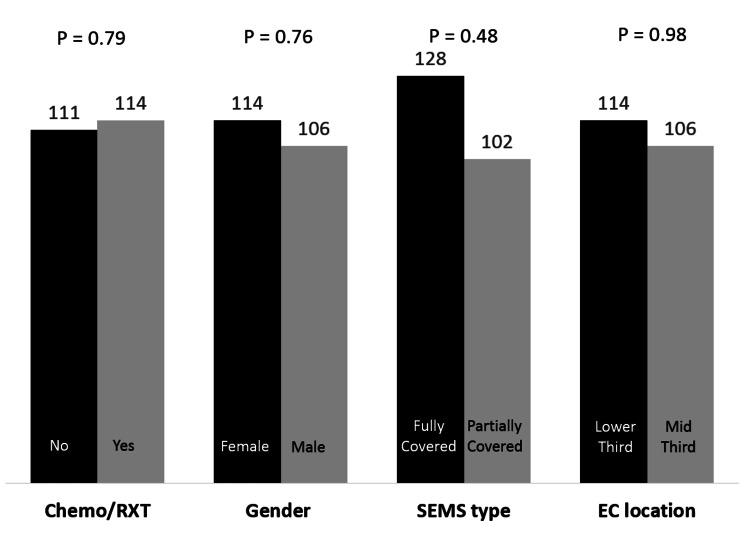
Median survival depending on chemo-radiotherapy, gender, stent type and tumor location

Survival data were available for 32 patients. The median survival of patient’s post SEMS placement was 114 days (range 30-498). EC located in mid third (median 126, range 30-458) and those who had a fully covered SEMS (median 128, range 45-298) survived longer, this however was not significantly different compared to EC in lower third (median 114, range 36-492) or partially covered SEMS (median 102, range 30-492) (p = 0.98 and p = 0.48, respectively). In addition, the survival in subgroup including chemotherapy/ radiotherapy or gender type did not differ significantly (Figure 3).

## Discussion

Our single tertiary referral center experience in this retrospective study demonstrated that stent placement for the inoperable malignant esophageal disease is effective. SEMS provided good palliation of dysphagia in all patients with symptomatic improvement in the ability to swallow without increasing the rate of complications compared to other studies.

The technical success of placing SEMS in our study was 100%, similar to the rates shown in most studies [10,11,17,20,22]. Even in studies that reported technical difficulties in stent placement the success rate was above 90% [4,7-9,19,21,23-25]. The reasons for failure were inadequate stent length, failure of stent deployment, and migration; these difficulties could be immediately overcome by either repositioning the stent or by placing another stent. Almost all patients in whom SEMS is successfully placed experience improvement in dysphagia.

However, the recurrence of dysphagia is a major drawback of SEMS and is seen in more than 50% of the cases and is reported more often in randomized than in other observational studies (Table 3). Tissue overgrowth has been consistently shown to be the major reason and could account for up to 90% of the recurrence [15]. The median time to development of tissue growth has been calculated to be around three months [6] and the incidence increases with longer survival and follow-up [2]. In addition, the specific design and larger diameter of the SEMS mitigate tissue growth [14,21]. Insertion of an overlapping SEMS or ablation of the excess tissue successfully restores patency with improvement in dysphagia. 20% of our study population developed tissue overgrowth which is in the mid-range of most retrospective studies which reported a range from 5% to 34% (Table 3).

**Table 3:** Complications in different studies classified according to design Tech suc: technical success; Dys imp: dysphagia improvement; Rec dys: recurrent dysphagia; Migrat: migration; Perf: perforation

Study Randomized	Stent type (n)	Tech suc (%)	Dys imp (%)	Rec dys (%)	Migrat (%)	Bleed (%)	Perf (%)	Tumor growth (%)	Survival (median days)
Randomized									
Verschuur et al., 2008 [11]	Partial (42)	100	100	52	17	12	0	31	132
	Complete (42)	90	100	31	12	5	0	24	159
Conio et al., 2007 [9]	Partial (54)	98	94	33	4	0	2	6	122
Sabharwal et al., 2003 [22]	Partial (31) (ultraflex)	100	100	10	6	3	0	3	NA
	Partial (22) (flamingo)	100	100	9	5	5	5	5	NA
Siersema, 2001 ( [10]	Partial (34) (ultraflex)	91	100	26	18	15	6	3	104
	Partial (33) (flamingo)	100	100	33	9	9	6	15	113
	Complete (31)	94	100	24	12	24	6	12	110
Kim, 2009 [26]	Complete (19)	100	100	63	5	11	0	26	62
	Complete (17) (layered)	94	100	12	0	0	6	0	74
Van Heel, 2012 [27]	Partial (40) (ultraflex)	100	100	40	8	18		2	NA
	Partial (40) (Evolution)	100	100	8	3	0		3	NA
Prospective									
Verschuur, 2007 [21]	Large dia (73)	95	100	14	3	15	5	7	100
	Small dia (265)	95	100	36	14	12	4	18	116
Homs, 2004 [14]	Partial (146)	NA	NA	32	15	NA	NA	13	NA
	Complete (70)	NA	NA	23	6	NA	NA	16	NA
Verschuur, 2006 [23]	Complete (42)	97	100	12	7	5	3	5	139
Talreja, 2012 [17]	Complete (37)	100	100		8	3			146*
Van Boeckel, 2010 [24]	Partial (44)	97	98	25	5	2	0	14	88
Uitdehaag, 2009 [19]	Complete (45)	100	100	49	36	4	0	16	146
Uitdehaag, 2010 [20]	Complete (44)	100	100	27	14	18	0	5	110
Walter, 2014 [25]	Complete (40)	98	98	23	15	8	0	5	76
Choi, ,2011 [28]	Complete (100)	100	100	19	6	2	0	7%	74
Repici, 2014 [16]	Complete (82)	99	100	23	12	4	1	9	144
Meike, 2012 [29]	Complete (28)	100	100	15	11	6	0	4	64
Tian, 2016 [18]	Complete (91)	100	100	4	5	7	0	0	128
Lazaraki, 2011 [15]	Complete (89)	93	92	43	5	0	0	39	202
White, 2009 [12]	Complete (951)	NA	96	NA	1	1	1.9	6	250
Van Boeckel. 2010 [30]	Partial (37)	97	100	22	6	0	0	10	116
Retrospective									
Ross, 2007 [7]	Partial (97)	NA	86	16	5	14	0	5	77
Sundelöf, 2007 [8]	Combination (149)	98	70	27	4	1	1	20	NA
Elphick, 2005 [3]	Combination (100)		100	24	1	15	NA	NA	52
Burstow M, 2009 [1]	Combination (90)	99	NA	NA	7	1	1	4	93
Eroglu, 2010 [4]	Partial (170)	99	100	28	10	6	1	18	177*
Mezes, 2014 [6]	Complete (56)	95	100	39	NA	7	0	34	127
Stewart, 2012 [31]	Combination (138)	NA	90	NA	2	2	1	12	90
Chen, 2015 [2]	Partial (92)	97	100	45	7	0	0	22	NA
	Partial (109) (modified)	96	100	29	10	1	0	11	NA

The other pertinent reason for dysphagia recurrence following SEMS is stent migration, with the reported rate varying between 2% to 18%. Although a significantly high incidence of migration (36%) was observed when using Alimaxx-E fully covered stent, which was speculated by the authors to be due to the specific design of the stent [19]. Some of the factors associated with a tendency towards higher migration rates included fully covered SEMS, smaller body diameter, histology of the tumor, type of stent, and ongoing therapy in form of chemoradiation [14,15,20,21,25]. Nevertheless, more than 90% of migrated stents could be endoscopically retrieved and the stricture was managed successfully by either repositioning or insertion of a new SEMS [14,20]. In this current study, the rate of migration was at the lower end of the range at 9%, comparable (4%-5%) to studies that used stents with similar design [7,8,24].

Major complications, e.g., bleeding and perforation are uncommon. The rate of bleeding reported in the literature is wide ranging from 0% to 24%. This may be due to the fact that the definition of bleeding is not standardized with some using just symptoms and others endoscopic evidence. The higher rate of bleeding has been related to the use of a specific stent design (Gianturo-Z/SX-ELLA stent) or the use of a larger diameter SEMS in the studies [10,20,21]. Perforation is less often encountered and is reported to vary from 0% to 4%. Dilatation of the malignant stricture prior to stenting results in more perforations and is no more a standard practice [10,21]. None of the SEMS inserted in our study were complicated with either bleeding or perforation. This may just be a reflection of the small sample size.

The strength of this study is the exclusive inclusion of only malignant strictures of the esophagus. Almost all previous studies included other causes like fistulas, external compressions, and bronchogenic malignancy in their analysis. This helps to better understand the performance of SEMS in this exclusive group. In addition, the SEMS insertion technique was standardized with all stents inserted endoscopically with fluoroscopic guidance without prior dilatation. On the contrary, in most studies, the stent insertion technique varied during the study period with some strictures being dilated prior and others inserted exclusively under fluoroscopy. This directly impacts the outcome and brings into question the endoscopic technique rather than the stent performance.

## Conclusions

In conclusion, SEMS placement is a successful tool in relieving symptoms of dysphagia in malignant esophageal strictures. Our study adds to the growing literature that SEMS insertion is feasible, effective, and safe. It is not associated with higher complication rates when performed without prior dilatation of the stricture. Recurrent dysphagia and stent migration are common but can be successfully managed endoscopically. Correct technique and the right choice of type of SEMS may play a major role in reducing the risk of complications. To better understand this, larger studies are needed to provide data that are more robust, and this may be best achieved by national registries.
